# Vitamin D3 mitigates myopathy and metabolic dysfunction in rats with metabolic syndrome: the potential role of dipeptidyl peptidase-4

**DOI:** 10.1007/s00210-024-03439-3

**Published:** 2024-10-02

**Authors:** Nourhan O. Shoier, Salah A. Ghareib, Hend Kothayer, Amira Ebrahim Alsemeh, Shaimaa S. El-Sayed

**Affiliations:** 1 Pharmacology and Toxicology Department, Faculty of Pharmacy, Zagazig University, Zagazig, 44519 Egypt; 2 Medicinal Chemistry Department, Faculty of Pharmacy, Zagazig University, Zagazig, Egypt; 3 Human Anatomy and Embryology Department, Faculty of Medicine, Zagazig University, Zagazig, Egypt

**Keywords:** Vitamin D3, Metabolic syndrome, Myopathy, DPP-4 inhibitors, Vildagliptin, Skeletal muscles

## Abstract

Metabolic syndrome is associated with vitamin D3 deficiency. This work aims to examine the efficacy of vitamin D3 in inhibiting MetS-induced myopathy and to determine whether the beneficial effects of vitamin D3 are mediated by the inhibition of dipeptidyl peptidase-4 (DPP-4). An in silico study investigated the potential effectiveness of vitamin D3 on the inhibition of the DPP-4 enzyme. An in vitro assay of the DPP-4 inhibitory effect of vitamin D3 was performed. In vivo and over 12 weeks, both diet (with 3% salt) and drinking water (with 10% fructose) were utilized to induce MetS. In the seventh week, rats received either vitamin D3, vildagliptin, a combination of both, or vehicles. Serum lipids, adipokines, glycemic indices, and glucagon-like peptide-1 (GLP-1), muscular glucose transporter type-4 (GLUT-4) content, DPP-4, adenosine monophosphate kinase (AMPK) activities, and Sudan Black B-stained lipids were assessed. Muscular reactive oxygen species (ROS), caspase-3, and desmin immunostaining were used to determine myopathy. MetS-induced metabolic dysfunction was ameliorated by vitamin D3, which also reduced intramuscular glycogen and lipid accumulation. This is demonstrated by the attenuation of MetS-induced myopathy by vitamin D3, decreased oxidative stress, increased desmin immuno-expression, and caspase-3 activity. Our in silico data demonstrated that vitamin D3 is capable of inhibiting DPP-4, which is further supported by biochemical findings. Vitamin D3 increased serum GLP-1, muscular AMPK activity, and GLUT-4 content, whereas the levels of muscular ROS were decreased in MetS. Vildagliptin and its combination with vitamin D3 yielded comparable results. It is suggested that the DPP-4 inhibitory potential of vitamin D3 is responsible for the amelioration of MetS-induced metabolic changes and myopathy.

## Introduction

Metabolic syndrome (MetS) refers to a set of metabolic risk factors that ultimately contribute to the development of diabetes and cardiovascular disease (Alberti et al. 2009). Obesity, hypertension, hypertriglyceridemia, hyperglycemia, and a decrease in high-density lipoprotein (HDL) are all relevant components of MetS (Reaven 1988). MetS is diagnosed when a patient exhibits at least three symptoms (Grundy et al. 2005). In addition, MetS and its complications have been linked to a Western-style diet characterized by high carbohydrates (such as fructose) and high salt intake (Lim et al. 2010). Noteworthy, salt can exacerbate the cardiovascular detrimental effects of fructose (Levanovich et al. 2021). Therefore, an animal model with high salt and high fructose is commonly used in experimental studies to replicate the outcomes of MetS in humans (Chan et al. 2021).

A significant proportion of body mass is comprised of skeletal muscles, which are the most prevalent insulin-sensitive tissue responsible for maintaining glucose homeostasis (Sylow et al. 2021). Accordingly, skeletal muscle insulin resistance (IR) plays a crucial role in metabolic dysregulation associated with MetS (Wu and Ballantyne 2017). In obesity, the intramyocellular lipid content of skeletal muscle increases, which leads to mitochondrial dysfunction and impaired muscle protein synthesis by interfering with the incorporation of amino acids into muscle proteins through lipotoxic effects (Masgrau et al. 2012). The metabolism of lipids in skeletal muscle and vitamin D3 has been shown to be significantly correlated, as vitamin D3 scarcity results in an increase in fatty infiltration in skeletal muscle, which in turn leads to skeletal muscle weakness and a decrease in muscle strength (Gilsanz et al. 2010). Furthermore, the reduced insulin signaling in skeletal muscles that is associated with MetS may be attributable to augmented inflammatory adipokines, glycogen accumulation, increased reactive oxygen species (ROS), and vitamin D3 deficiency (Szymczak-Pajor and Śliwińska 2019). The inflammatory response induces modifications in the intermediate filaments of skeletal muscle, which leads to adipocyte accumulation and a delay in skeletal muscle regeneration (Paulin and Li 2004). Moreover, vitamin D3 deficiency affects muscular contractility by reducing Ca^+2^ entrance into the sarcoplasmic reticulum, prolonging the relaxation phase (Dzik and Kaczor 2019), and reducing adenosine monophosphate-activated protein kinase (AMPK) activity that is linked to skeletal muscle IR (Chang and Kim 2019). Conversely, vitamin D3 supplementation alleviates oxidative stress and activates AMPK in myocytes, reversing MetS-induced IR (Li et al. 2022).

Gliptins are inhibitors for dipeptidyl peptidase-4 (DPP-4), which have been demonstrated to enhance insulin sensitivity and provide significant pleiotropic effects. These effects are primarily achieved by elevating the level of glucagon-like peptide-1 (GLP-1), which subsequently binds to its receptors in the pancreas, thereby stimulating insulin secretion. This blocking of glucagon release leads to a reduction in hepatic glucose production (Ahrén et al. 2011). However, the role of gliptins in preventing MetS-associated progression of myopathy has largely been overlooked.

A DPP-4 inhibitory action of vitamin D3 and its active metabolites has not been investigated. Therefore, we conducted an in silico computational study to determine the DPP-4 inhibitory potentials of vitamin D3 and its metabolites at the molecular level. In addition, these potentials were examined in vitro by assessment of the DPP-4 inhibitory effect of vitamin D3 and in vivo by utilizing a well-established animal model of MetS-induced myopathy (3% salt in diet and 10% fructose in water for 12 weeks). The molecular underlying mechanisms implicated in vitamin D3-mediated amelioration of MetS-induced metabolic derangement and myopathy were also examined and compared to a well-established DPP-4 inhibitor, vildagliptin. Moreover, the effect of combined vildagliptin-vitamin D3 on MetS-induced myopathy and metabolic derangement was investigated.

## Material and methods

### Molecular modelling

In order to rationalize the DPP-4 inhibitory effect of vitamin D3 (cholecalciferol) at the molecular level, a docking study was performed for vitamin D3 (cholecalciferol) and its activated metabolites calcifediol (25-hydroxycholecalciferol, 25-OH vitamin D3) and calcitriol (1,25-dihydroxycholecalciferol, 1,25-(OH)2 vitamin D3) on the crystal structure of complex of human DPP-4 and inhibitor (BI 1356, linagliptin) (PDB ID:2RGU) (Eckhardt et al. 2007). This was accomplished using the Molecular Operating Environment (MOE) version MOE 2019.0102 (Chemical Computing Group, Montreal, CA) (ULC, 2021); Chemical Computing Group Inc.: Montreal). The co-crystal structure of the DPP-4 enzyme with inhibitor-encoded PDB ID: 2RGU was downloaded from the RCSB protein data bank (PDB) website (http://www.rcsb.org) (Eckhardt et al. 2007). The proteins were processed prior to docking using the MOE quick preparation tool. All the water molecules, other heteroatoms, and repeated chains were removed. Vitamin D3 and its activated metabolites (calcifediol and calcitriol) preparation were prepared by minimizing energy, adding hydrogen atoms, calculating partial charges and potential energy, and using the previously described docking protocol (Kothayer et al. 2019). Validation was achieved by replicating the original orientation when redocking the co-crystallized inhibitor within the enzyme’s binding site (score − 9.9171, RMSD 1.7147 Å). The best-docked confirmation was determined using the docking score and interactions generated from the output. Vitamin D3 and its activated metabolites (calcifediol and calcitriol) were subjected to a flexible alignment study with the co-crystalized inhibitor (BI 1356, linagliptin) of DDP-4 (PDB ID:2RGU) and vildagliptin using MOE 2019.0102. The obtained conformations (S; Kcal/mol) were evaluated using the configuration alignment score.

### *In vitro *assay of DPP-4 inhibitory effect

To validate the results of the in silico screen and directly assess the inhibitory effect of vitamin D3 on DPP-4, we used a DPP-4 inhibitor screening assay kit obtained from Abcam (ab133081, MA, USA). The assay procedures included serial dilutions of vitamin D3 (2, 3.9, 7.8, 15.63, 31.25, 62.5, 125, and 250 µM) and compared them to a positive control DPP-4 inhibitor (sitagliptin at a concentration of 100 µM). Samples were assayed in triplicates, and all procedures were performed following the manufacturer’s instructions. Finally, the percentage inhibition of DPP-4 activity was calculated against the vehicle control (no inhibitor wells).

### Animals

Thirty adult male Wistar albino rat‏s (weighing 150–200 g) were obtained from the animal house of the Faculty of Veterinary Medicine, Zagazig University, Egypt. The rats were housed in the animal care unit at the Faculty of Pharmacy, Zagazig University, Egypt. Throughout the course of the study, rats were kept in plastic cages (four rats/cage) under typical light/dark cycle conditions (12/12 h), temperature (23 ± 2 °C), and humidity (60 ± 10%). Rats were fed a standard chow diet and tap water ad libitum.

### Drugs and chemicals

Vildagliptin was offered as a gift by Eva Pharma Company (Cairo, Egypt), vitamin D3 (type 100 CWS) was purchased from Sigma Pharmaceutical Company (Menoufia, Egypt), and sodium chloride was from El-Nasr Pharmaceutical Chemicals Company (Sharqia, Egypt) while fructose was from Specialized Food Industries-Safety Misr Company (Sharqia, Egypt). All chemicals used were at high purity.

### Metabolic syndrome (MetS) induction and experimental protocol

MetS-induction was initiated in 24 rats after the acclimatization period by administering high fructose (10%) in drinking water and high salt (3%) in diet (HFrHS) for 12 weeks (Rault-Nania et al. 2008). The control group (Group 1) consisted of the remnant rats (*n* = 6) who were maintained on a standard rat diet and tap water for the duration of the study. The establishment of MetS was tested by challenging rats with an oral glucose tolerance test (OGTT) and fasting serum lipid profile measurement 6 weeks after the initiation of the special diet (HFrHS). Rats with confirmed MetS were randomly assigned to one of four groups (2–5), *n* = 6 per group. The rats were then administered drugs or vehicles for 6 weeks while being maintained on an HFrHS diet. Group 2 received the vehicle and served as MetS-group. Group 3 rats acquired vitamin D3 orally by gavage (10 µg/kg/day). Group 4 rats received vildagliptin orally by a gavage (10 mg/kg/day). Group 5 rats received both vildagliptin (10 mg/kg/day) and vitamin D3 (10 µg/kg/day) orally, initiated with vildagliptin followed by vitamin D3 (2 h apart) (Wahba et al. 2021).

### Non-invasive blood pressure (NIBP) recording

Two days before the completion of the study, blood pressure was recorded in conscious rats utilizing the tail-cuff technique (Li et al. 2011). Briefly, the recordings were conducted using a power lab system from AD Instruments (Bella Vista, Australia). The system was equipped with an NIBP pulse transducer (NIBP3047020, Pan lab HA® Harvard Apparatus Ltd, Kent, UK) that was connected to a computer operating the professional software lab chart (version 8).

### Measurement of anthropometric parameters

Rats’ body weight (BW), length (from nose to anus), and waist circumference (WC) were all evaluated at the conclusion of the study. These measurements were then used to estimate the values for body mass index (BMI) by dividing BW (g) over length square (cm^2^) (Allah and Abdelrahman 2021).

### Blood and tissue sampling

After the experiment concluded and an overnight fast, blood sampling was conducted under anesthesia with thiopental sodium (50 mg/kg, i.p) (Helmy et al. 2019). Blood was withdrawn from the retro-orbital plexus in heparinized tubes, allowed to clot for 30 min at 4°C, and subsequently centrifuged at 4000 r.p.m. for 15 min at 4 °C. The aspirated serum was stored at − 80 °C for subsequent analysis. Euthanasia was confirmed using an overdose of thiopental sodium (100 mg/kg), followed by decapitation (Helmy et al. 2019). Soleus muscle was isolated, instantly frozen with liquid nitrogen, and kept at − 80 °C for further examination. Visceral fats (mesenteric and retroperitoneal) were dissected and weighed, then divided by BW.

### Biochemical analysis

#### Determination of metabolic parameters (glycemic control, uric acid, lipid profile, adipokines)

Serum glucose concentration was measured utilizing a colorimetric kit (BioMed-Glucose) provided by Egy Chem (GLU109480, Badr City, Egypt), following the manufacturer’s instructions. For OGTT, overnight fasting animals were challenged with a glucose solution (20%, 2 g/kg, gavage). Blood sampling was performed before glucose administration and at 30, 60, and 120 min following oral glucose load (Khanal et al. 2010). Soleus muscle glycogen contents were determined using a colorimetric kit from LSBio (LS-K183, WA, USA). Serum insulin level was measured in fasting rats using a rat ELISA kit from Crystal Chem’s (90010, IL, USA). As an indication of insulin resistance, the homeostasis model assessment of insulin resistance (HOMA-IR) was derived from fasting serum insulin (FSI) in µU/mL and fasting serum glucose (FSG) in mg/dL using the formula: HOMA-IR = (FSG × FSI)/22.5 (Er et al. 2016). Fasting serum hemoglobin A1c (HbA1c) level was determined using a rat ELISA Kit provided by My BioSource (MBS2033689, CA, USA), while serum uric acid was assessed using a colorimetric kit obtained from Spinreact (MD41001, Spain). All assays were performed following the manufacturer’s instructions.

Serum triglycerides (TG), high-density lipoprotein cholesterol (HDL-C), and total cholesterol (TC) were determined using colorimetric kits (MD41031, MD1001096, and TK41021, respectively, Spinreact, Spain), as per the manufacturer’s instructions. These measurements were used to calculate serum low-density lipoprotein cholesterol (LDL-C) according to the equation LDL-C = TC – (HDL-C + TG/5) (Friedewald et al. 1972) and atherogenic index (AI) following the equation AI = (TC-HDL-C)/HDL-C (Li et al. 2021). Serum leptin and adiponectin levels were determined using rat ELISA kits from Crystal Chem (90040 and 80570, respectively, IL, USA), according to the manufacturer’s instructions.

#### Determination of serum GLP-1, soleus muscle dipeptidyl peptidase-4 (DPP-4) and AMPK activities, and glucose transporter type 4 (GLUT-4) level

The serum GLP-1 level was measured using a rat ELISA kit provided by Elabscience Biotechnology (E-EL-R0059, TX, USA). A fluorometric kit provided by Abnova (KA3737, Taipei, Taiwan) was utilized to assess DPP-4 activity in soleus muscle homogenate. In this kit, DPP-4 cleaves a substrate (proline-containing peptide, H-Gly-Pro-AMC) to release a quenched fluorescent group, AMC (7-Amino-4-Methyl Coumarin) (Ex/Em = 360/460 nm). Additionally, the AMPK activity of soleus muscle homogenate was assessed using an immunoassay kit provided by MBL Life Science (CY-1182, Nagano, Japan). The GLUT-4 level was determined using a rat ELISA kit provided by CUSABIO (CSB-E13908r, TX, USA), and all measurements followed the manufacturer’s instructions.

#### Determination of AGEs, oxidative stress (NADPH Oxidase, ROS), and apoptotic markers (caspase-3 activity) in soleus muscle

AGEs, NADPH oxidase, and ROS were quantified in soleus muscle homogenate using rat ELISA kits from MyBioSource (MBS700464, MBS2602768, and MBS039665, respectively, CA, USA). In contrast, the caspase-3 activity in soleus muscle homogenate was assessed using a colorimetric kit purchased from BioVision (K106-25, CA, USA). All measurements were conducted in accordance with the manufacturer’s instructions.

#### Determination of serum 1,25(OH)_2_D3 levels

Serum 1,25(OH)_2_ D3 level was determined using a rat ELISA kit obtained from LSBio (LS-F27932-1, WA, USA) following the manufacturer’s guidelines.

### Immunohistochemical staining

Five-micrometer-thick sections were cut from paraffinized blocks of soleus muscle for immunohistochemical staining. Briefly, the sections were immersed in a citrate buffer with a concentration of 10 mM and a pH of 6.0. Sections were heated in a water bath at a temperature of 98 °C for 30 min and subsequently rinsed with water. Following that, the sections were treated with H_2_O_2_ (3% in methanol) for 15 min to block endogenous peroxidase activity. In order to block non-specific binding, sections were incubated for 10 min with horse serum at room temperature. Subsequently, the primary rabbit polyclonal antibody for desmin (1:200, # 602–300, AbboMax, San Jose, USA) was applied to the prepared sections, and they were incubated at 4 °C overnight. Subsequently, sections were incubated with biotinylated secondary antibody, then avidin–biotin complex (Vectastain® ABC-peroxidase kit, Vector Laboratories, Burlingame, CA, USA). The color was generated by adding 3,3-diaminobenzidine (DAB) solution to the sections. Images of the prepared sections were captured using a bright-light microscope (LEICA ICC50 W) in the Image Analysis Unit of the Anatomy and Embryology Department. ImageJ software plugin and immunohistochemistry (IHC) profiler were used to determine the percentage of positive brown stained areas as per the previously explained method (Varghese et al. 2014). The results represent the mean ± standard error of the mean (SEM) data obtained from three sections/animal (*n* = 3 rats/group).

### Histochemistry of lipids in soleus muscle

Total lipid staining with Sudan Black B (Sigma-Aldrich Corp, St. Louis, MO, USA) was performed on 5-µm-thick soleus muscle sections following the method described by Chiffelle and Putt (1951). Briefly, Sudan Black B solution was formed by decomposing 1 g of the powder in 70 ml isopropyl alcohol for 30 min, followed by the addition of 30 ml of distilled water containing 0.3 g dextrin. The final solution was allowed to stand for 1 day and then filtered just before use. Sections were deparaffinized, hydrated in 70% alcohol, air dried, and immersed into Sudan Black B solution for at least 1 h. Afterward, sections were rinsed thoroughly in two changes of 70% alcohol, followed by rinsing with six changes of distilled water. The prepared sections were then mounted with glycerin jelly, and images were captured using a bright-light microscope (LEICA ICC50 W) in the Image Analysis Unit of the Anatomy and Embryology Department.

### Statistical analysis

Data are displayed as mean ± SEM. Statistical analysis was performed using GraphPad Prism (version 9.1.0 (221), GraphPad Software Inc., CA, USA). One-way analysis of variance (ANOVA) and post hoc Tukey’s test were used for comparisons. Statistical significance was determined at *p* < *0.05*.

## Results

### Molecular modeling findings

**Fig. 1:**
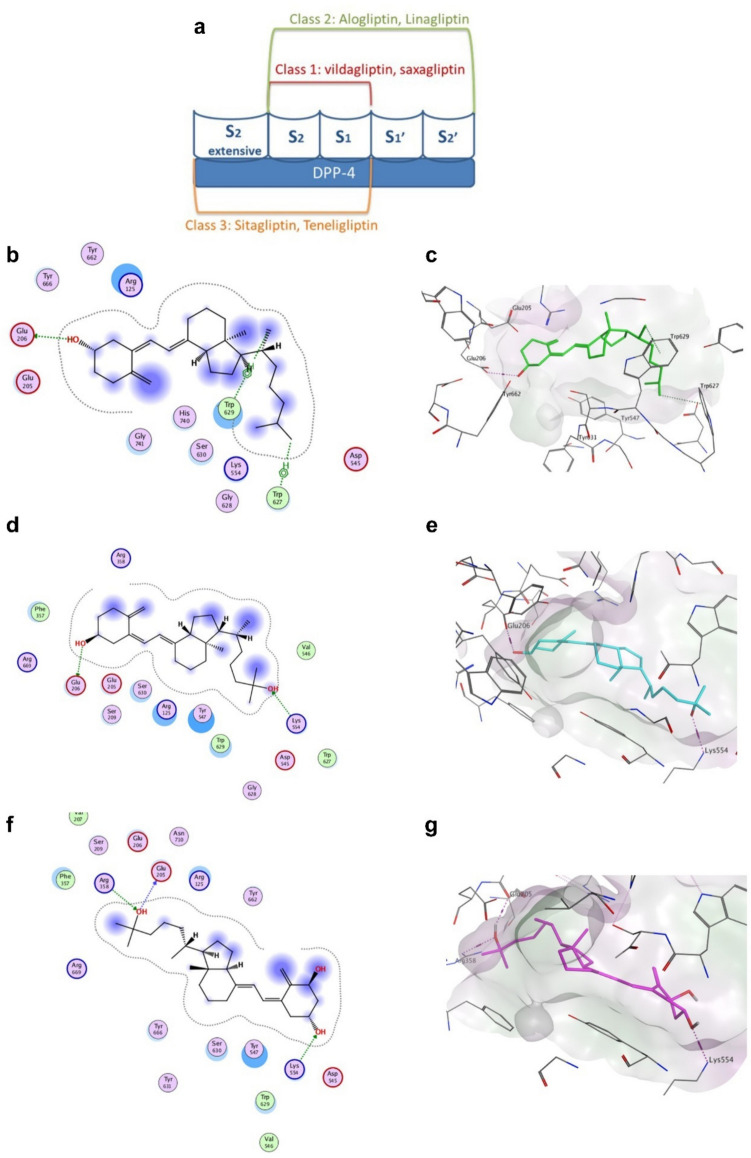
The binding subsites of the various classes of dipeptidyl peptidase-4 (DPP-4) inhibitors into the DPP-4 active site (**a**). Docking poses of vitamin D3 or its metabolites with DPP-4 (PDB ID:2RGU) are shown, where panels **b** and **c** show 2D and 3D docking poses of cholecalciferol (green), panels **d** and **e** show 2D and 3D docking poses of calcifediol (cyan). In contrast, panels **f** and **g** show 2D and 3D docking poses of calcitriol (purple)

**Fig. 2:**
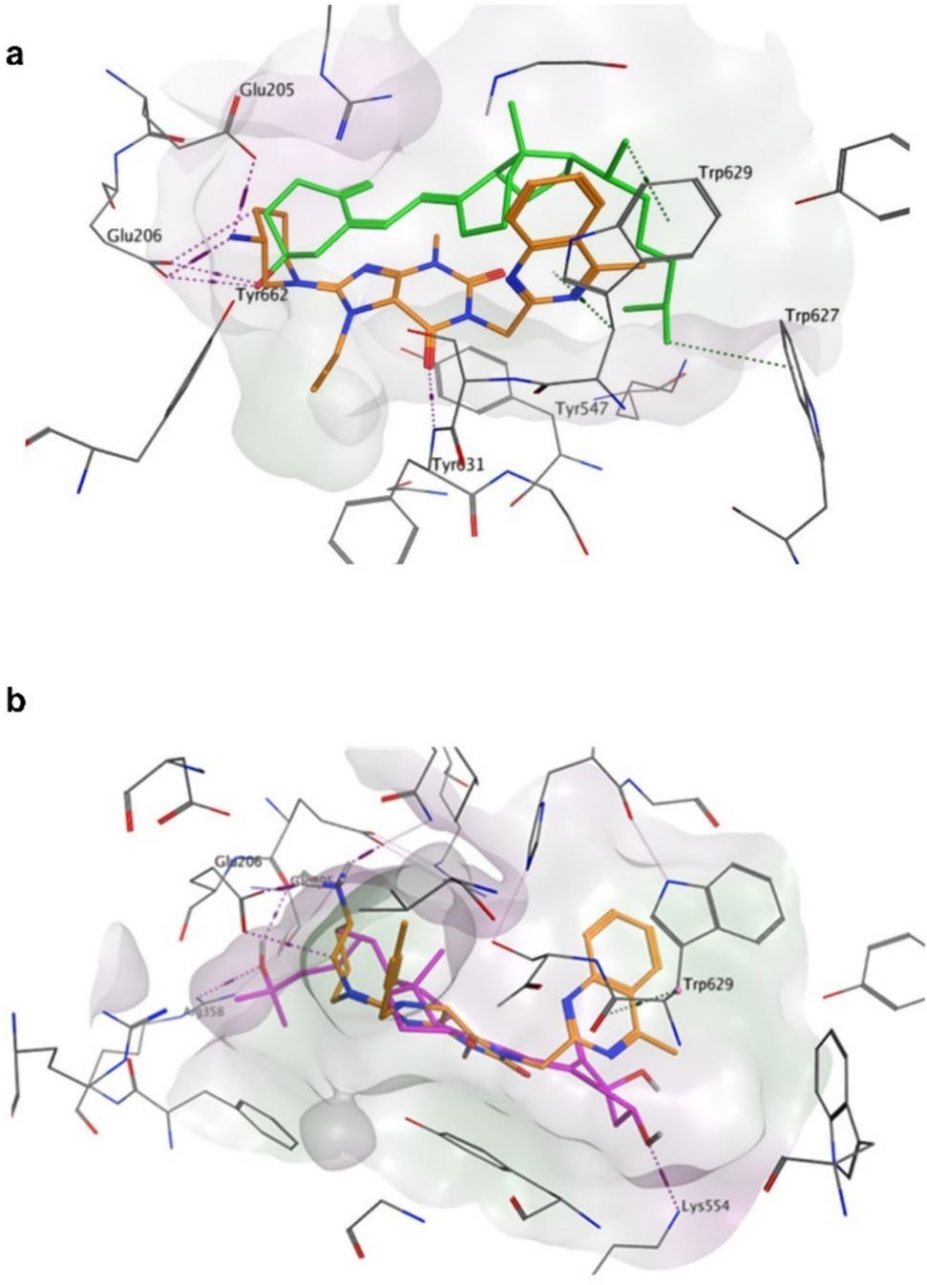
3D binding interaction pattern of vitamin D3 (green, panel **a**) and calcitriol (purple, panel **b**) in the binding site of DPP-4 (PBD: 2RGU). The binding orientation of these compounds is similar to that of the co-crystalized inhibitor (BI 1356, linagliptin) shown in orange

The active site of DPP-4 involves S1, S2, S1', S2', and S2 expansive subsites modified from previous studies (Nabeno et al. 2013, Maladkar et al. 2016) which are depicted in Fig. 1a. The binding modes of cholecalciferol, calcifediol, and calcitriol (scores of − 6.5338, − 7.0530, and − 8.1767, respectively) demonstrate that they all successfully bind to either Glu205 or Glu206 in the S2 subsite via a hydrogen bond, as evidenced by all of the DPP-4 inhibitors (Figs. 1b and c and 2a). Additionally, calcifediol and calcitriol succeeded in occupying the S2' subsite (like linagliptin) but this time by forming a hydrogen bond with Lys554 (Figs. 1d and g and 2b). Furthermore, calcitriol formed an extra hydrogen bond interaction with Arg358 in the S2 extensive subsite (Figs. 1f and g and 2b).

**Fig. 3:**
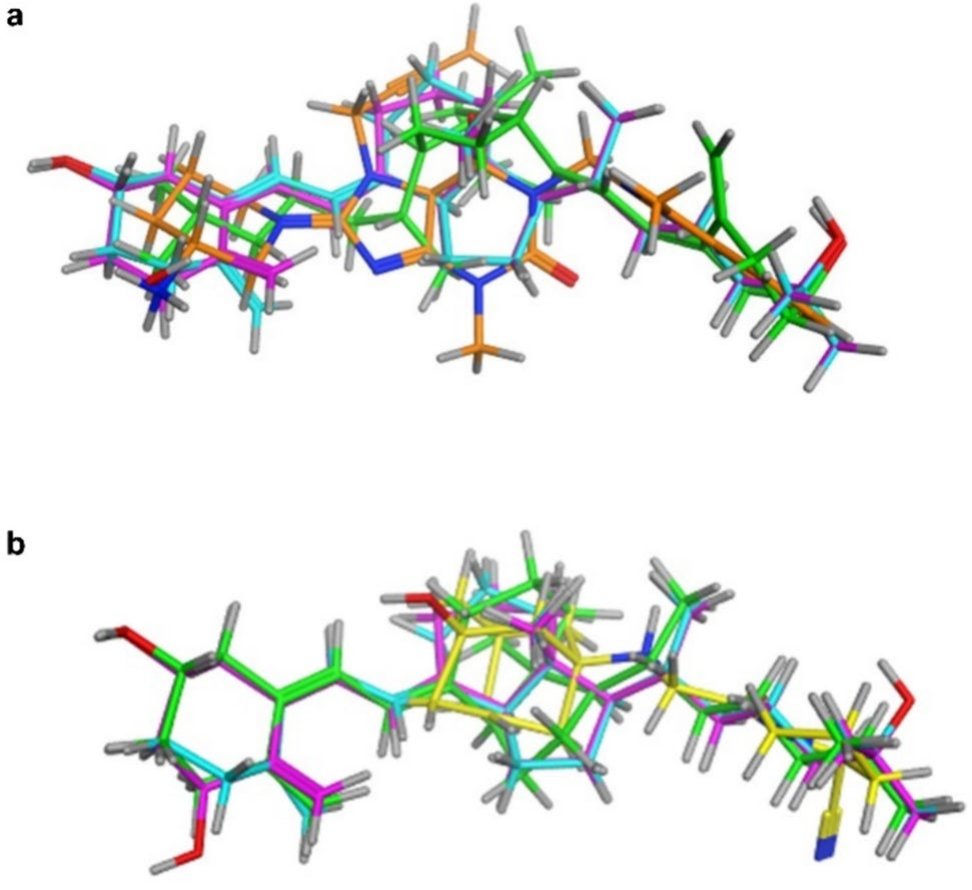
Flexible alignment of cholecalciferol (green), calcifediol (cyan), and calcitriol (purple) with the co-crystalized inhibitor (BI 1356, linagliptin) (orange) of DDP-4 (PDB ID:2RGU) (**a**) and the vildagliptin (yellow) (**b**)

Moreover, the structural similarities between cholecalciferol, calcifediol, calcitriol, and linagliptin were examined using an adaptable alignment approach. The 3D flexible alignment analysis revealed that vitamin D3 and its metabolites exhibited conformations that were comparable to those of linagliptin. The configuration alignment score (S) is − 76.0479 kcal/mol, which suggests a state of perfect alignment (Fig. 3a). Nevertheless when cholecalciferol, calcifediol, and calcitriol were examined for their structural similarities to vildagliptin using the adaptable alignment technique, the findings indicated a low similarity to vildagliptin. The configuration alignment score (S) was − 19.2878 kcal/mol, indicating a poor alignment (Fig. 3b).

### Effect of vitamin D3 on DPP-4 enzyme activity *in vitro*

Vitamin D3 exhibited a dose-related inhibition of DPP-4 activity, as illustrated in Table 1. A pronounced inhibition was detected at a concentration of 62.5 µM, with the highest concentration (250 µM) approaching the inhibitory percentage of DPP-4 activity in the positive control (Sitagliptin).

**Table 1:** Enzymatic inhibition of DPP-4 activity after treatment with vehicle, sitagliptin, and vitamin D3

Sample	Concentration	DPP-4 activity inhibition (%)
Vehicle		0
Sitagliptin	100 µM	88.163
Vitamin D3	2 µM	21.613****
	3.9 µM	23.691****
	7.8 µM	23.833****
	15.6 µM	26.028****
	31.25 µM	29.756****
	62.5 µM	70.402****
	125 µM	78.146****
	250 µM	81.258***

Data are presented as mean ± SEM (*n* = 3/sample). One-way ANOVA followed by Tukey’s test for multiple comparisons was employed (comparison to sitagliptin, ****p < 0.0001 and ***p < 0.001)

### Effect on blood pressure

**Fig. 4:**
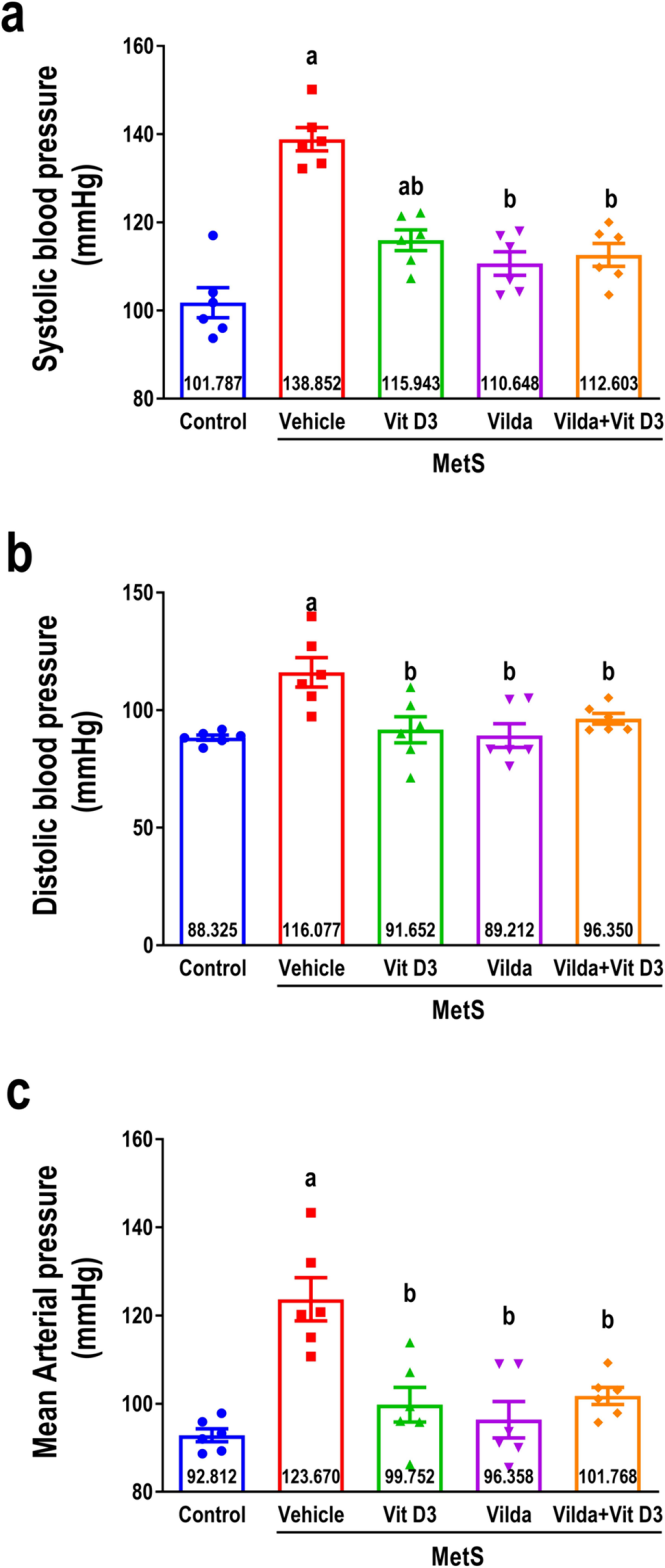
Effect of 6 weeks of treatment with vitamin D3 (10 µg/kg/day, gavage), vildagliptin (Vilda,10 mg/kg/day, gavage), or combination on metabolic syndrome (MetS)-induced changes in systolic blood pressure (**a**), diastolic blood pressure (**b**), and mean arterial pressure (**c**). MetS was induced by feeding rats 3% salt and 10% fructose for 12 weeks. Values are presented as mean ± SEM (*n* = 6/group). One-way ANOVA followed by Tukey’s test for multiple comparisons was used for analysis, ^a^ vs control and ^b^ vs MetS at *p* < *0.05*

As demonstrated in (Fig. 4), MetS rats exhibited a marked elevation in systolic blood pressure (SBP), diastolic blood pressure (DBP), and mean arterial pressure (MAP) (Fig. 4a, b, and c, respectively) compared to the control group indicating hypertension. Hypertension in MetS rats was significantly alleviated upon treatment with vitamin D3, as demonstrated by a significant reduction in SBP, DBP, and MAP compared to the vehicle-treated MetS rats. Comparable findings were observed with the administration of vildagliptin and the combined administration compared to the vehicle-treated MetS rats.

### Effect on anthropometrical parameters

**Fig. 5:**
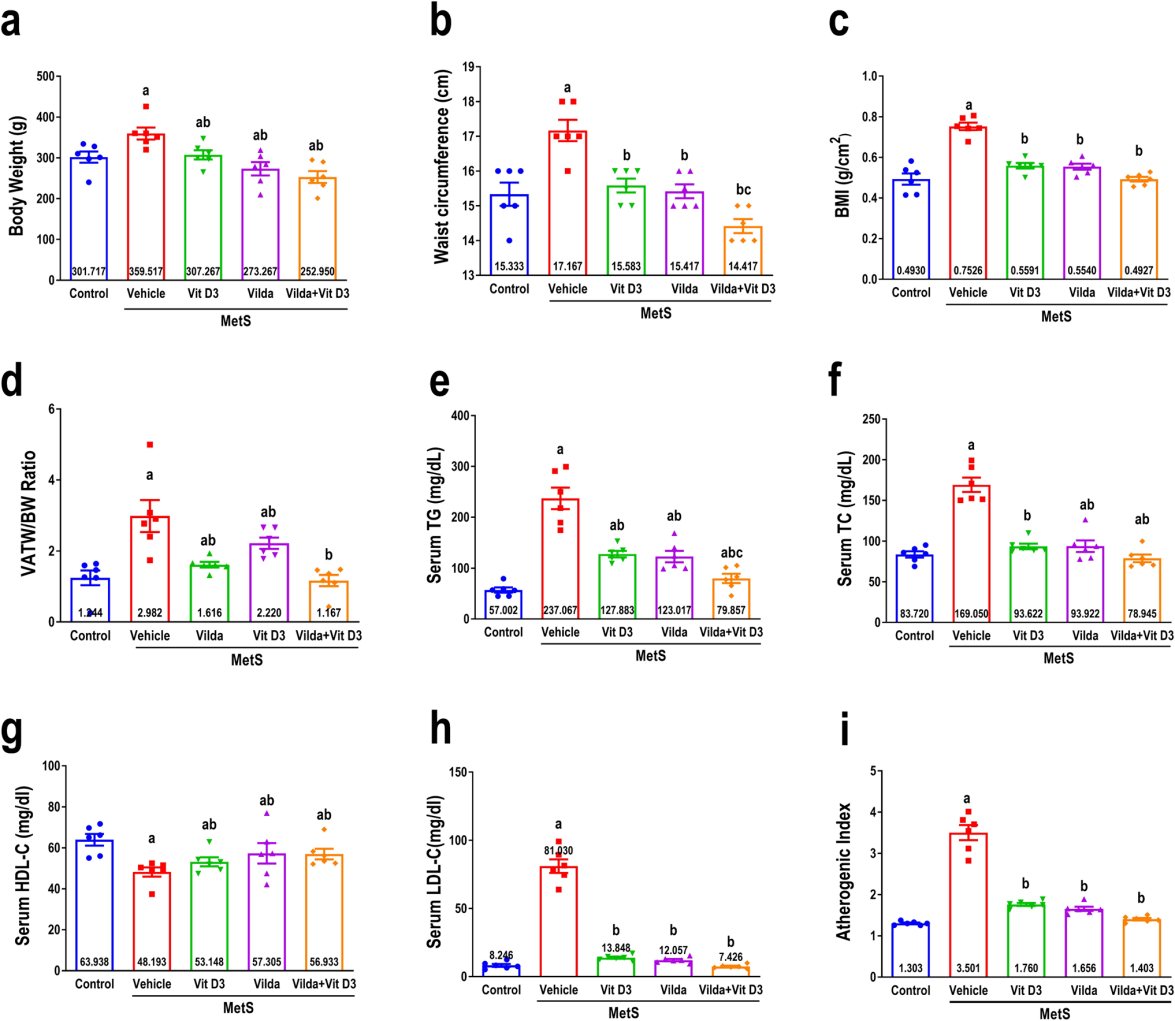
Effect of 6 weeks of treatment with vitamin D3 (10 µg/kg/day, gavage), vildagliptin (Vilda,10 mg/kg/day, gavage), or combination on metabolic syndrome (MetS)-induced anthropometrics alterations as expressed by body weight (BW, **a**), waist circumference (WC, **b**), body mass index (BMI, **c**), and visceral adipose tissue weight/bodyweight (VATW/BW, **d**). They also demonstrated their effect influence on MetS-induced disturbances of lipid profile, as manifested by serum levels of triglycerides (TG, **e**), total cholesterol (TC, **f**), high-density lipoprotein-cholesterol (HDL-C, **g**), low-density lipoprotein-cholesterol (LDL-C, **h**), and atherogenic index (**i**). MetS was induced by feeding rats 3% salt and 10% fructose for 12 weeks. Values are presented as mean ± SEM (*n* = 6/group). One-way ANOVA, followed by Tukey’s test for multiple comparisons, were used for analysis, ^**a**^ vs control, ^b^ vs MetS, and.^c^ vs vildagliptin at *p* < *0.05*

As depicted in Fig. 5, MetS rats exhibited significant disruption of anthropometrics as evidenced by weight gain and augmentation in WC, BMI, and VATW/BW ratio (Fig. 5a, b, c, and d, respectively) compared to the control group. By significantly reducing BW, WC, BMI, and VATW/BW ratio in comparison to the vehicle-treated rats with MetS, vitamin D3 and vildagliptin significantly improved anthropometrical parameters. Simultaneously, their combination exhibited a significant improvement in anthropometrics comparable to the MetS group and further showed a notable decline in WC compared to vildagliptin alone.

### Effect on lipid profile

As demonstrated in Fig. 5, MetS rats exhibited a marked elevation in serum TG, TC, and LDL-C as well as atherogenic (Fig. 5e, f, h, and i, respectively) while presenting a significant reduction in HDL-C (Fig. 5g) compared to the control group indicating as remarkable hyperlipidemic status. Serum lipid derangements in MetS rats were significantly alleviated upon treatment with vitamin D3. This was demonstrated by a significant reduction in TG, TC, LDL, and AI and considerable augmentation of HDL-C contrasted with untreated MetS rats. Comparable findings were observed when vildagliptin administration and combined administration were used compared to the untreated MetS rats. Interestingly, the dual administration of both drugs resulted in a remarkable decline in TG compared to vildagliptin treatment.

### Effect on glycemic control

**Fig. 6:**
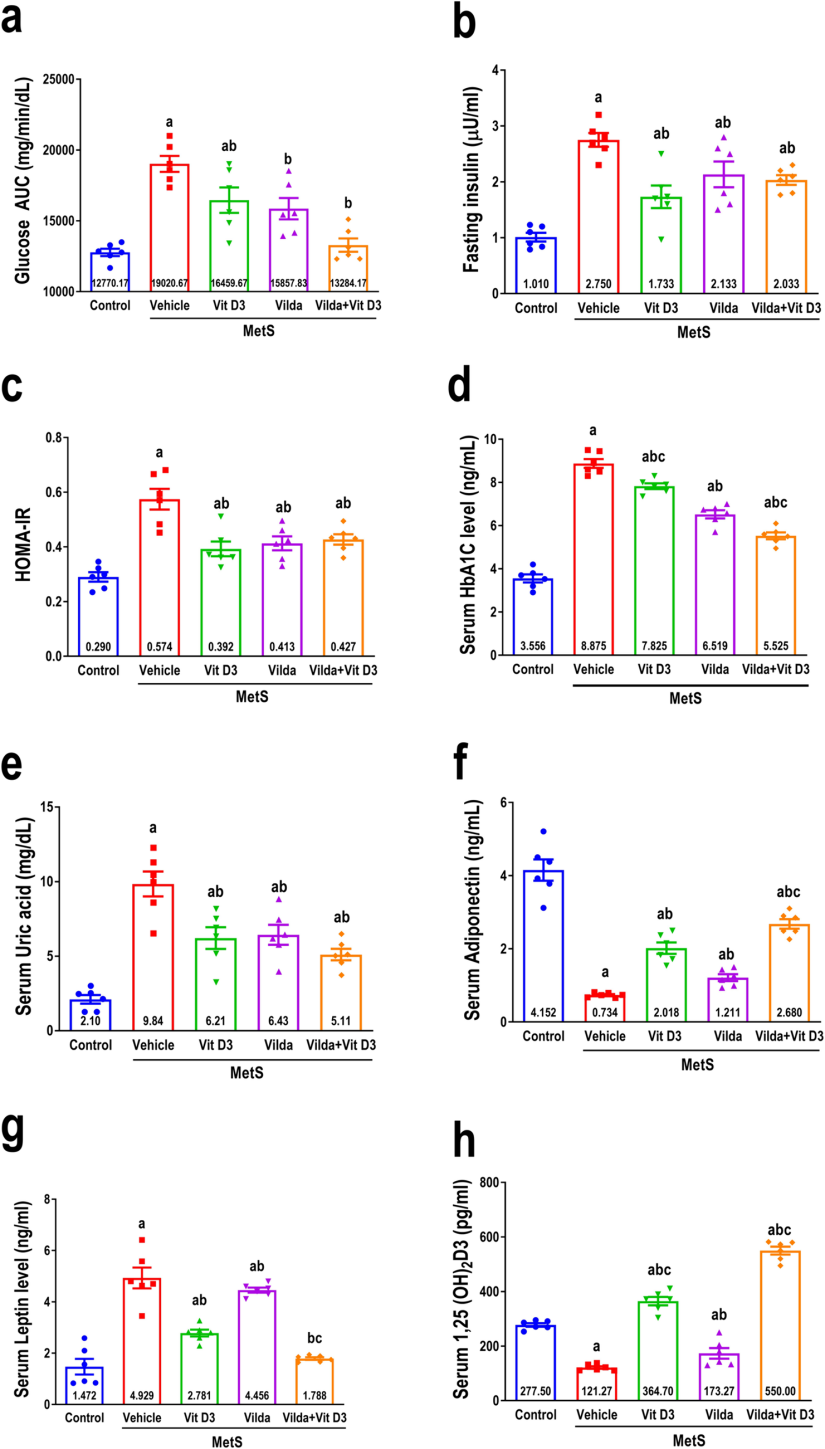
The effect of 6 weeks of treatment with vitamin D3 (10 µg/kg/day, gavage), vildagliptin (Vilda, 10 mg/kg/day, gavage), or combination on metabolic syndrome (MetS)-induced impairment of glycemic control demonstrated by the area under glycemic curve of oral glucose tolerance test (AUC of OGTT, **a**), fasting insulin (**b**), HOMA-IR (**c**), and HbA1C (**d**). Additionally, they demonstrated their effect on MetS-induced changes in serum uric acid (**e**) and serum adipokines (adiponectin, **f**, and leptin, **g**) as well as vitamin D3 deficiency (1,25(OH)_2_D3, **h**). MetS was induced by feeding rats 3% salt and 10% fructose for 12 weeks. Values are presented as mean ± SEM (*n* = 6/group). One-way ANOVA, followed by Tukey’s test for multiple comparisons, was used for analysis, ^a^ vs control, ^b^ vs MetS, and.^c^ vs vildagliptin at *p* < *0.05*

The glycemic parameters of MetS rats were significantly impaired, as evidenced by a significant increase in the area under the glycemic curve constructed from OGTT, fasting serum insulin, HOMA-IR, and serum HbA1C, as shown in Fig. 6a to d. Vitamin D3 significantly improved glycemic control by attenuating MetS-induced effects in a manner similar to vildagliptin. However, it was equivalent to the vildagliptin-treated MetS group, as vitamin D3 exhibited a significantly higher HbA1C (1.2-fold). Although the combination was not superior to individual treatments, this group exhibited a 15% greater decrease in HbA1C levels than the vildagliptin treatment.

### Effect on serum uric acid, adipokines, and 1,25(OH)_2_D3

MetS rats exhibited a significant decrease in both serum adiponectin (Fig. 6g) and 1,25(OH)_2_D3 (Fig. 6i). In contrast, there is a substantial increase in both serum leptin (Fig. 6h) and uric acid (Fig. 6f) compared to the control group. Vitamin D3 treatment exhibited a 2.8-fold significant augmentation in serum adiponectin and a threefold considerable elevation in serum 1,25(OH)_2_D3 level. In contrast, serum leptin and serum uric acid significantly decreased compared to the vehicle-treated MetS rats. Similar to the treatment with vildagliptin, the serum adiponectin and 1,25(OH)2D3 levels are significantly increased (1.7- and onefold, respectively). Conversely, the serum leptin and serum uric acid levels were significantly reduced compared to those of the vehicle-treated MetS rats. Additionally, the combination of vildagliptin and vitamin D3 led to a significant elevation in serum adiponectin and serum 1,25(OH)2D3 levels (1.7- and onefold, respectively). In contrast, a substantial decrease in serum leptin level and serum uric acid was seen compared to the vehicle-treated MetS rats. Moreover, the combination of vildagliptin and vitamin D3 resulted in significant elevation (in serum adiponectin level and serum 1,25(OH)_2_D3 level, 3.5- and 4.5-fold, respectively, compared the untreated MetS rats and 2- and 4.5-fold, respectively, in contrast to vildagliptin alone. However, the combination significantly reduced serum leptin and serum uric acid levels compared to vehicle-treated MetS rats.

### Effect glycogen contents and fat deposition in soleus muscle

**Fig. 7:**
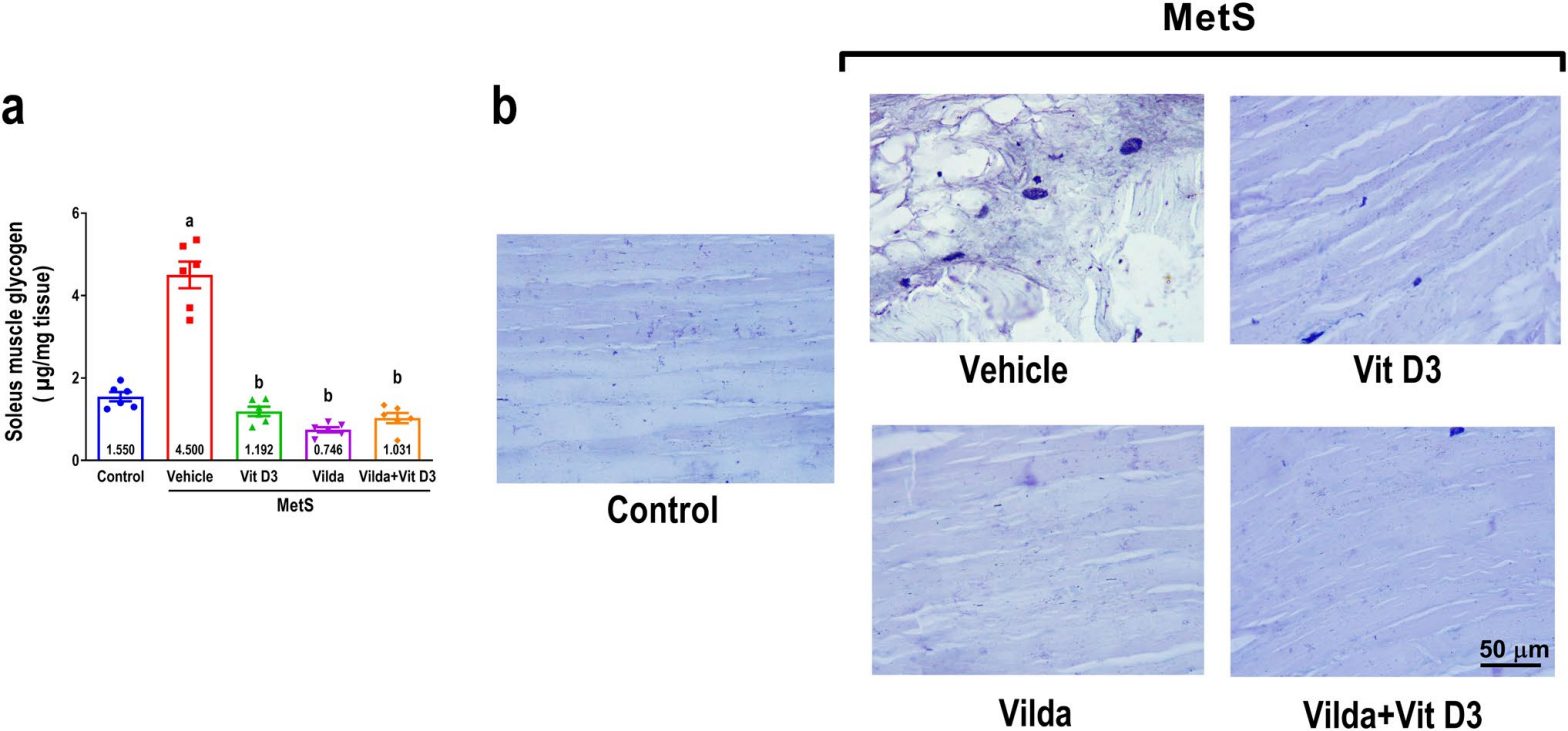
The effect of 6 weeks treatment with vitamin D3 (10 µg/kg/day, gavage), vildagliptin (Vilda,10 mg/kg/day, gavage), or combination on metabolic syndrome (MetS)-induced glycogen accumulation (**a**). MetS was induced by feeding rats 3% salt and 10% fructose for 12 weeks. Values are presented as mean ± SEM (*n* = 6/group). One-way ANOVA, followed by Tukey’s test for multiple comparisons, were used for analysis, ^a^ vs control, ^b^ vs MetS, and.^c^ vs vildagliptin at *p* < *0.05*. Panel **b** represents photomicrographs of Sudan Black staining for total lipids of the longitudinal sections from the soleus muscle (*n* = 3/group). Scale bar 50 µm × 400

As demonstrated in Fig. 7a, MetS rats exhibited a notable accumulation of glycogen in the soleus muscle (threefold) compared to the control group, indicating impaired glycogen breakdown. Vitamin D3, vildagliptin, and their combination significantly declined soleus muscle glycogen content compared to the treated MetS group. Similarly, as presented in Fig. 7b, MetS rats exhibited increased fat deposition in soleus muscle sections stained with Sudan Black B staining, where numerous lipid droplets were seen. In contrast, the administration of either vitamin D3, vildagliptin, or both exhibited a decreased number of lipid droplets.

### Effect on serum GLP-1 and soleus muscle DPP-4, AMPK activity, and GLUT-4 levels

**Fig. 8:**
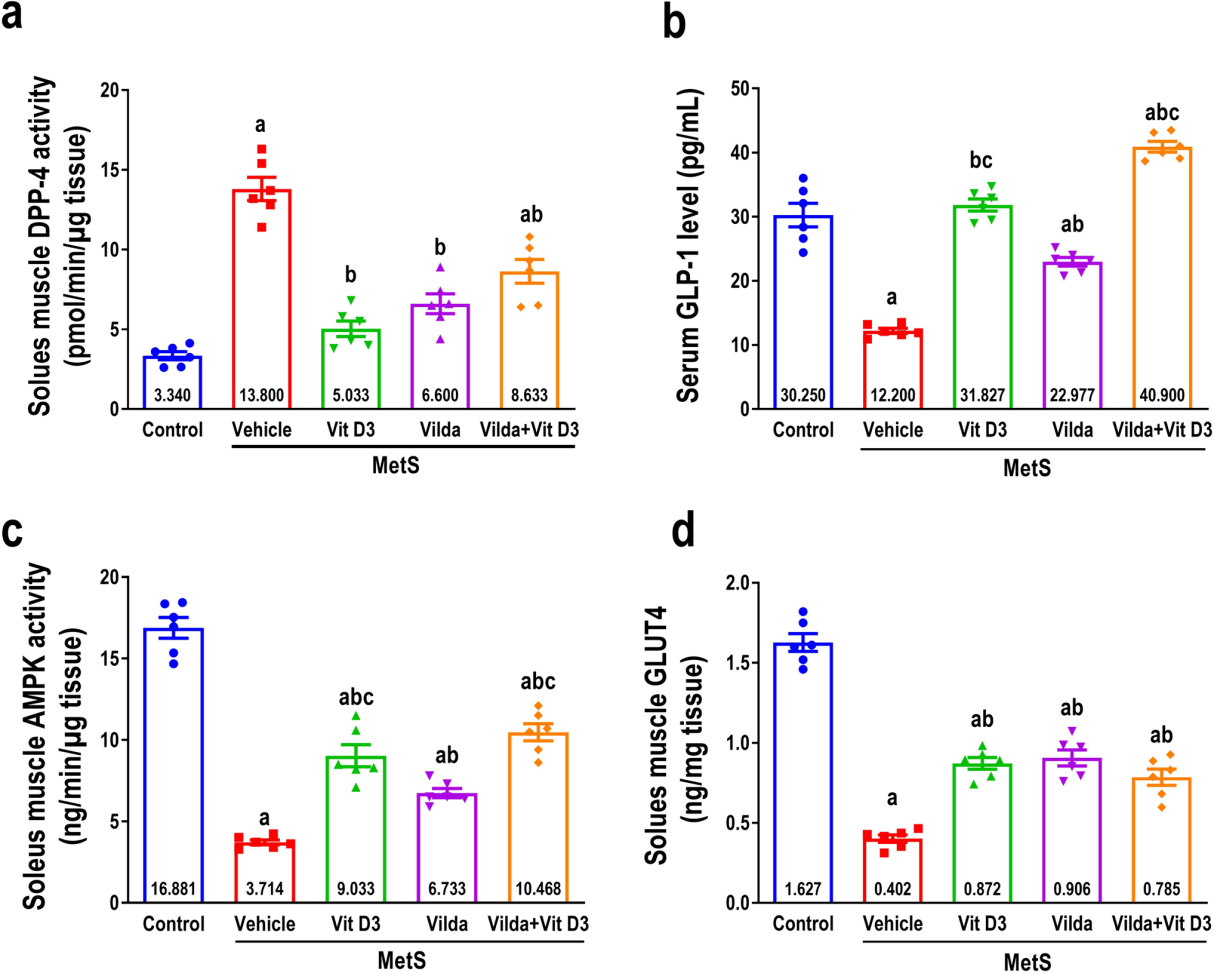
The effect of 6 weeks of treatment with vitamin D3 (10 µg/kg/day, gavage), vildagliptin (Vilda,10 mg/kg/day, gavage), or combination on metabolic syndrome (MetS)-induced changes in soleus muscle DPP-4 activity (**a**), serum GLP-1 (**b**), soleus muscle AMPK activity (**c**), and soleus muscle GLUT-4 (**d**). MetS was induced by feeding rats 3% salt and 10% fructose for 12 weeks. Values are presented as mean ± SEM (*n* = 6/group). One-way ANOVA, followed by Tukey’s test for multiple comparisons, was used for analysis, ^a^ vs control, ^b^ vs MetS, and.^c^ vs vildagliptin at *p* < *0.05*

As demonstrated in Fig. 8a–d, MetS rats exhibited a significant fourfold augmentation in DPP-4 activity in the soleus muscle. However, there was a significant decrease in serum GLP-1 level, AMPK activity, and GLUT-4 content in the soleus muscle compared to the control group. Vitamin D3 treatment exhibited a substantial decrease in soleus muscle DDP-4 activity compared to vehicle-treated MetS rats, consistent with our molecular modeling predictions and in vitro screening results. This effect was similar to that of vildagliptin. Nevertheless, the combination appeared less effective than the individual drug effects. Additionally, the serum GLP-1 level, AMPK activity, and GLUT-4 levels in soleus muscle were significantly elevated compared to those of vehicle-treated MetS rats by vitamin D3, vildagliptin, or their combination. Vitamin D3 significantly increased the AMPK activity of the soleus muscle and the serum GLP-1 level compared to the vildagliptin treatment, with a threefold and 1.5-fold increase, respectively. Similarly, the combination elevated considerably soleus muscle AMPK activity (1.5-fold) and serum GLP-1 (1.8-fold) compared to vildagliptin alone.

### Effect on soleus muscle oxidative stress and apoptotic markers

**Fig. 9:**
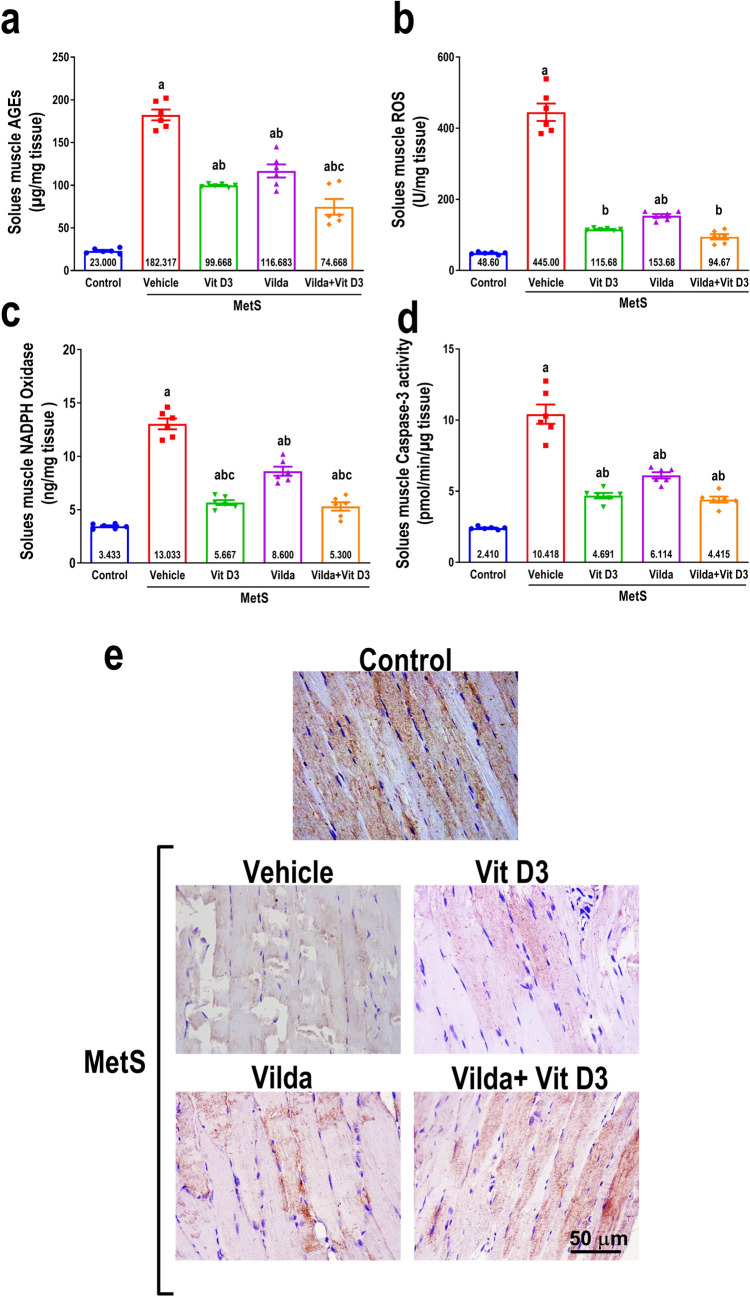
The effect of 6 weeks treatment with vitamin D3 (10 µg/kg/day, gavage), vildagliptin (Vilda, 10 mg/kg/day, gavage), or combination on metabolic syndrome (MetS)-induced myopathy expressed as soleus muscle advanced glycation end products (AGEs, **a**), reactive oxygen species (ROS, **b**), nicotinamide adenine dinucleotide phosphate oxidase (NADPH Oxidase, **c**), and caspase-3 activity (**d**). MetS was induced by feeding rats 3% salt and 10% fructose for 12 weeks. Values are presented as mean ± SEM (*n* = 6/group). One-way ANOVA, followed by Tukey’s test for multiple comparisons, was used for analysis, ^a^ vs control, ^b^ vs MetS, and.^c^ vs vildagliptin at *p* < *0.05*. Panel **e** represents photomicrographs of immunohistochemical staining of desmin in the longitudinal section of rat soleus muscle sections from study groups (n = 3/group) (Scale bar 50 µm × 400)

As presented in Fig. 9a–d, MetS rats exhibited significant augmentation in oxidative stress markers (AGEs, NADPH Oxidase, and ROS) and proapoptotic markers (caspase-3 activity) in the soleus muscle, compared to the control group. Vitamin D3 alleviated oxidative stress and apoptosis in soleus muscle, as demonstrated by a significant decline in AGEs, NADPH Oxidase, ROS, and caspase-3 activity compared to the untreated MetS rats. Similar findings were observed upon vildagliptin and its combination with vitamin D3. NADPH oxidase was significantly reduced by vitamin D3 administration in comparison to vildagliptin alone (5.66 ± 0.19 vs 8.6 ± 0.34). Conversely, when administered in conjunction with vildagliptin, it significantly decreased AGEs and NADPH oxidase (74.67 ± 10.96 vs 116.7 ± 10.74 and 5.3 ± 0.33 vs 8.6 ± 0.34, respectively).

### Immunohistochemical staining findings

The immunohistochemical staining of soleus muscle sections for desmin revealed a strong striated staining pattern in the control group. In contrast, the treated MetS group revealed weak positive immuno-expression, indicating myopathy. The immuno-expression of desmin was further enhanced by the administration of vitamin D3, vildagliptin, or a combination of both, with the combination showing a relatively similar effect (Fig. 9e).

## Discussion

The Western-style diet, characterized by high fructose/high salt (HFrHs), directly affects the development of MetS and associated diseases (Abdelhedi et al. 2019). The majority of total body glucose utilization is attributed to muscles. Therefore, their malfunction predisposes them to insulin resistance (IR) (Yu et al. 2012). Muscle loss, or intramuscular lipid accumulation, is a hallmark of metabolic dysfunction (Collins et al. 2018). Consequently, myopathy may arise due to risk factors for MetS. Furthermore, myopathy has not been extensively investigated, even though cardiovascular diseases and type 2 diabetes have garnered significant attention as detrimental effects of MetS.

Vitamin D3 has been shown to be beneficial in the treatment of MetS-related ailments (Ferreira et al. 2020). The effects of vitamin D3 through DPP-4 inhibition require further investigation. An in silico investigation was performed to determine the DPP-4-inhibitory potentials of vitamin D3 and its metabolites at the molecular level. This was further validated by in vitro screening of vitamin D3-induced inhibition of DPP-4 activity by testing several concentrations of vitamin D3 and comparing it to the positive control, sitagliptin. Moreover, an animal model of MetS-induced myopathy (3% salt, 10% fructose for 12 weeks) was used. Additionally, the molecular processes involved in the effects of vitamin D3, such as the inhibition of DPP-4, were examined in comparison to that of vildagliptin. The selection of soleus muscle in this study was based on its susceptibility to metabolic and oxidative alterations in response to diet change (Bozkurt et al. 2010; Pinho et al. 2017).

Weight gain and visceral obesity are characteristic symptoms of MetS (Gordish et al. 2017), which supports our findings. Although most DPP-4 inhibitors have no considerable effects on weight (Gilbert and Pratley 2020), vildagliptin resulted in a modest weight loss herein, which might be attributable to its intestinal fat extraction inhibition (Matikainen et al. 2006). Vitamin D3 exhibited comparable effects to vildagliptin on anthropometric measurements, which aligns with prior findings on the negative correlation between serum vitamin D3 levels and anthropometric indicators (Baradaran et al. 2012; Kang et al. 2013). Prior research suggests that obesity predisposes to vitamin D3 deficiency (Muscogiuri et al. 2010), while others reported vitamin D3 insufficiency as a risk variable for MetS (Moy and Bulgiba 2011). Vitamin D3 deficiency is linked to an increased risk of MetS ailments, including hypertriglyceridemia, lower HDL-C levels, and high LDL-C (Melguizo-Rodríguez et al. 2021). This finding is consistent with our reported findings that HFrHS-induced MetS was associated with diminished calcitriol and HDL-C serum levels, while hypertriglyceridemia and high LDL-C were found. The observed effect was reversed with the use of vitamin D3, which is consistent with a prior study that documented a significant decrease in triglyceride and LDL-C levels when vitamin D3 was administered (Ghaderi et al. 2017). Similar findings were observed with vildagliptin, which agrees with earlier research demonstrating lipid profile improvement with vildagliptin in high-fat diet-fed mice (Zhou 2019) and in type 2 diabetic patients (Shimodaira et al. 2015).

Hypertension is another feature of MetS that is strongly associated with its pathogenesis, including obesity, and represents a major risk factor for cardiovascular morbidity and mortality associated with MetS (Franklin 2006; Mohamed et al. 2024), which is consistent with our findings. In the present study, vitamin D3 demonstrated a blood pressure-lowering effect, which can be attributed to the inhibition of RAAS (Pashova-Stoyanova and Tolekova 2019), improvement of endothelial cell function, and decrease in atherosclerosis risks (Sona 2022). Furthermore, vildagliptin, either alone or combined with vitamin D3, elicited an antihypertensive effect, which is consistent with previous studies where the blood pressure-regulating effect of vildagliptin was attributed to modulating vascular endothelial growth factor levels and improving physiological angiogenesis and vasculature, in addition to improving the lipid profile (Wu et al. 2017b; El-Naggar et al. 2019).

In this study, poor glycemic control may be attributable to pancreatic β-cell dysfunction, which might result from MetS-associated vitamin D3 deficiency as it impairs β-cell capability to activate pro-insulin, thus influencing insulin secretion and sensitivity predisposing to IR (Oh and Barrett-Connor 2002). Hyperinsulinemia, elevated HbA1C, and HOMA-IR were established herein, indicating IR alleviated with vitamin D3, vildagliptin, or a combination of both, consistent with prior studies (Wahba et al. 2020, 2021). Robust data indicates a strong correlation between the intake of fructose and the development of hyperuricemia (Zhang et al. 2020). Our findings were supported by the reduction of 1-α hydroxylase suppressing calcitriol levels (Chen et al. 2014). Moreover, vitamin D3 decreases hyperuricemia in MetS rats, most likely by increasing uric acid clearance (Nimitphong et al. 2021), and comparable effects were reported with vildagliptin (Wahba et al. 2020).

Visceral adipose tissue mass changes alter several inflammatory cytokines, and adipokines are crucial in skeletal muscle IR development (Esposito and Giugliano 2004). The HFr/HS model augmented leptin while reducing adiponectin serum levels. Vitamin D3 modulates blood leptin and adiponectin (Mai et al. 2017), whereas vildagliptin directly affects adipocytes, decreasing leptin release while increasing adiponectin (Wei et al. 2021). Hyperleptinemia has been shown to decrease insulin-stimulated glucose uptake by inhibiting the activation of p38 mitogen-activated protein kinase and the translocation of GLUT-4 in response to insulin (Sweeney et al. 2001). Impaired adiponectin may improve IR in skeletal muscle by decreasing the uptake and oxidation of fatty acids, which is necessary to activate AMPK and translocate GLUT-4 to the cell surface (Liu and Liu 2014).

In this study, the HFr/HS model augmented soleus muscle DPP-4 activity, with a significant decline in serum GLP-1. This finding aligns with a previous study that revealed induced skeletal muscle DPP-4 activity on fructose consumption, which can modulate β-cell function and decrease serum GLP-1 level (Chen et al. 2019). Moreover, enhanced DPP-4 activity accelerated GLP-1 clearance and inhibited its indication in skeletal muscle tissue, precipitating inflammation and apoptosis and contributing to MetS-induced myopathy (Wahba et al. 2020). GLP-1 inhibits the first signaling pathways that cause skeletal muscle glucotoxicity and oxidative stress. Furthermore, it decreased the levels of GLP-1 and increased the glucotoxicity in skeletal muscle, partly due to impeded activation of AMPK and downregulated GLUT-4 translocation (Berger et al. 2018) (Umek et al. 2021), which agrees with our findings.

The current study used computational docking to establish the inhibitory potentials of vitamin D3 or its metabolites on DPP-4. The binding of all DPP-4 inhibitors to Glu205 and Glu206 in the S2 subsite is essential for their inhibitory action (Nabeno et al. 2013; Li et al. 2018; Kumar et al. 2021). The inhibitory effect of specific inhibitors can be significantly improved by binding to sites beyond the S2 subsite (Yoshida et al. 2012). Due to its interaction with Trp629 in the S2’ subsite, linagliptin has an eightfold more pronounced effect than alogliptin (Nabeno et al. 2013). The level of DPP-4 inhibition demonstrates a positive correlation with the number of coupling subsites. In addition to the crucial interactions with the S1 and S2 subsites, further associations with the S1’, S2’, or S2 extended subsites may enhance DPP-4 inhibition more effectively than forming a covalent bond with Ser630 (Nabeno et al. 2013). The present investigation demonstrated the effective interaction of vitamin D3 (cholecalciferol), calcifediol, and calcitriol with Glu205 or Glu206 in the S2 subsite, similar to all DPP-4 inhibitors. This interaction follows a hydrogen bonding mechanism and involves additional interactions with Trp629 or Lys554 in the S2’ subsite, similar to linagliptin. In addition, calcitriol expanded upon the S2 extensive subsite by forming hydrogen bonds with Arg358.

Furthermore, vildagliptin is a covalent inhibitor of DPP-4 that specifically binds to the S2 pocket by forming a covalent bond with Ser630 through its nitrile group (Berger et al. 2018). The absence of covalent bonding groups in the structures of vitamin D3 or its metabolites is thought to result in a distinct binding interaction pattern compared to that of vildagliptin. Our docking and flexible alignment studies revealed favored binding interactions of vitamin D3 and its metabolites into the DPP-4 active site with a binding pattern comparable to that of linagliptin, and this could explain the DPP-4 inhibitory activity of vitamin D3. These results were corroborated by the direct suppression of DPP-4 activity in laboratory settings by vitamin D3, which occurred in a manner dependent on the concentration. Additionally, the reduction of DPP-4 activity in our in vivo model replicated the effects of a DPP-4 inhibitor. In addition, the suggested direct suppression of DPP-4 by vitamin D3 and the observed weight loss and decreased cholesterol levels resulting from vitamin D3 treatment in this study may provide further insight into the downregulation of DPP4 activity. This finding aligns with a previous study on bariatric surgery (Herz et al. 2021).

In addition to metabolic dysfunction, there were observable morphological indications of muscle atrophy, including the evident absence of transverse striations in skeletal muscle fibers and elevated intramyocellular lipid content, consistent with previous studies (Umek et al. 2021). Lipid accumulation contributes to the toxic effect of MetS on muscle development (Hua et al. 2017). A potential explanation for the glycogen accumulation observed in the MetS group in this work is the downregulation of glycogen phosphorylase by fructose-1-phosphatase (Cambri et al. 2014). In high fructose models, glycogen accumulation is attributable to the inhibition of glycogen degradation rather than increased synthesis (Youn et al. 1987). Vitamin D3, vildagliptin, or both reversed these MetS-elicited effects. Vitamin D3 improved glucose utilization via stimulating AMPK activity/GLUT4 translocation and glycogen degradation in skeletal muscle (Manna et al. 2017). Recent reports suggest that DPP-4 inhibitors may mitigate the effects of MetS-induced myopathy by inducing skeletal muscle fatty acid oxidation and intramyocellular lipid reduction (Boschmann et al. 2009). Our observations, consistent with previous studies, indicate that vitamin D3 supplementation causes a significant intramyocellular lipid reduction, eliminates extra fat accumulation, and maintains muscle reliability (Romeu Montenegro et al. 2021), supporting our hypothesis regarding DPP-4 inhibitory potentials.

Furthermore, MetS-induced myopathy might be explained by increased muscular oxidative stress and apoptosis. AMPK-inactivation upregulated NADPH oxidase and enhanced ROS production, precipitating oxidative stress-associated myopathy (Ferreira and Laitano 2016). Hyperuricemia is associated with upregulation of skeletal muscle AGEs (Mastrocola et al. 2016). Accumulated AGEs cause oxidative stress, which triggers inflammatory and fibrotic processes in skeletal muscles and enhances oxidative stress and myopathy (Yamagishi 2011; Boyer et al. 2015). In this study, MetS enhanced caspases-3 activity, which may be attributable to AGE accumulation, which triggers proapoptotic proteins (Bax and caspase-3) (Kluck et al. 1999; Varghese et al. 2003). Vitamin D3 ameliorated muscular oxidative stress by downregulating NADPH oxidase and ROS, congruent with prior studies (Manna et al. 2017). Vildagliptin consistently demonstrated findings similar to previous investigations (Zhan et al. 2012; Wu et al. 2017a). The inhibitory effect of vildagliptin on MetS-induced muscular apoptotic injury may be attributed to its ability to enhance GLP-1, which in turn activates anti-apoptotic (Bcl-2) cells, suppresses Bax expression, and reverses caspase-3 activation (Zhan et al. 2012). Interestingly, vitamin D3 elicited similar findings and was added to vildagliptin on AGEs and caspase-3 activity suppression in skeletal muscle, concurs with a previous study that showed vitamin D3-induced inhibition of AGEs accumulation (Salum et al. 2013).

Obesity is a persistent low-grade inflammation that increases the risk of developing MetS. Therefore, the inflammatory response contributes to skeletal muscle extracellular matrix remodeling, predisposing to skeletal muscle mechano-signal transduction alteration mediated by intermediate filaments such as desmin. Ultimately, this leads to altered sensing of contractile activity and reduced mitochondrial function and number (Coletta and Mandarino 2011). The study demonstrated that vitamin D3, vildagliptin, or a combination markedly increased the desmin level in skeletal muscle tissue, thus alleviating MetS-induced myopathy.

## Conclusion

Vitamin D3 attenuated MetS-related metabolic abnormalities and associated myopathy. The effects of vitamin D3 have been suggested to be partially mediated by its inhibitory mechanism of DPP-4, which is supported by the following evidence: (1) the results of molecular modeling showed that vitamin D3 binds effectively to the DPP-4 enzyme; (2) direct inhibition of DPP-4 activity in vitro by vitamin D3; and (3) vitamin D3 reproduced the ameliorative effects on MetS-induced metabolic defects and myopathy. The combined therapy was not more effective than the individual drugs. Nevertheless, vitamin D3 supplementation is recommended for managing MetS-associated vitamin D3 deficiency.

## Data Availability

Data is provided within the manuscript or supplementary information files.

## Abbreviations

**AGEs**: Advanced glycation end products
**AMPK**: Adenosine monophosphate-activated protein kinase
**AUC**: Area under the curve
**1,25(OH)**_**2**_**D3**: 1,25-Dihydroxy vitamin D3
**BMI**: Body mass index
**BW**: Body weight
**DPP-4**: Dipeptidyl peptidase-4
**FSG**: Fasting serum glucose
**FSI**: Fasting serum insulin
**GLP-1**: Glucagon-like peptide-1
**GLUT-4**: Glucose transporter type4
**HbA1c**: Hemoglobin A1c
**HDL-C**: High-density lipoprotein cholesterol
**HFrHS**: High fructose/high salt
**HOMA-IR**: Homeostasis model assessment of insulin resistance
**IR**: Insulin resistance
**LDL-C**: Low-density lipoprotein cholesterol
**MetS**: Metabolic syndrome
**OGTT**: Oral glucose tolerance test
**ROS**: Reactive oxygen species
**TC**: Total cholesterol
**TG**: Triglycerides
**WC**: Waist circumference
